# Prediction of Cyclic O_6_ Molecules Stabilized by Helium under Pressure

**DOI:** 10.1002/advs.202415517

**Published:** 2025-01-24

**Authors:** Jingyu Hou, Qiang Zhu, Xiao‐Ji Weng, Xi Shao, Xiao Dong, Hui‐Tian Wang, Xiang‐Feng Zhou, Yongjun Tian

**Affiliations:** ^1^ Center for High‐Pressure Science State Key Laboratory of Metastable Materials Science and Technology School of Science Yanshan University Qinhuangdao 066004 China; ^2^ Key Laboratory of Weak‐Light Nonlinear Photonics School of Physics Nankai University Tianjin 300071 China; ^3^ Department of Mechanical Engineering and Engineering Science University of North Carolina at Charlotte Charlotte NC 28223 USA; ^4^ National Laboratory of Solid‐State Microstructures School of Physics Collaborative Innovation Center of Advanced Microstructures Nanjing University Nanjing 210093 China

**Keywords:** cyclic molecules O_6_, helium‐bearing system, high pressure

## Abstract

Oxygen usually exists in the form of diatomic molecules at ambient conditions. At high pressure, it undergoes a series of phase transitions from diatomic O_2_ to O_8_ cluster and ultimately dissociates into a polymeric O_4_ spiral chain structure. Intriguingly, the commonly found cyclic hexameric molecules in other group VIA elements (e.g., S_6_ and Se_6_) are never reported in the bulk oxygen. Through extensive computational crystal structure search, herein it is reported that such hexameric O_6_ molecules can exist in a stable compound HeO_3_ above 1.9 TPa. The first‐principles calculations reveal that, during the reaction by mixing oxygen with helium, the insertion of helium does not only expand the lattice volume, but also relieves the electron lone pair repulsion among diatomic O_2_, and thus significantly promoting the formation of cyclic O_6_ molecules. Furthermore, the transition pathway calculations demonstrate that molecular O_2_ is dissociated first, and then six oxygen atoms form a polymeric digital 2‐shaped intermediate O_6_. Subsequently, each unstable intermediate O_6_ decomposes into two intermedia O_3_ trimers. Finally, O_3_ trimers transform into cyclic O_6_ molecules at high pressure. This study expands the known molecular forms of oxygen and suggests a route to the synthesis of intriguing cyclic O_6_ molecules.

## Graphical abstract

The cyclic O_6_ molecules may exist due to the presence of analogical S_6_ and Se_6_. However, such O_6_ rings are never achieved due to unfavorable energy. Strikingly, a stable compound of HeO_3_ is discovered under pressure, consisting of neutral He atoms and O_6_ molecules. This report fills the gap in the forms of oxygen and expands knowledge of oxygen dissociation.

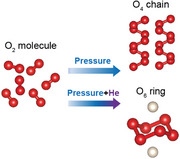

## 1 Introduction

Oxygen is the third most abundant element in the universe and indispensable to life. Investigations of the structures and properties of oxygen are essential for both fundamental science and technological applications. Owing to its unique magnetism, oxygen has been found to possess a rich phase diagram and many intriguing properties.^[^ ^1^ ^]^ Oxygen usually exists as a gas of diatomic molecules at ambient conditions. It undergoes a series of magnetic phase transitions as temperature decreases, including the paramagnetic γ‐phase at 54.39 K, the magnetically disordered β‐phase at 43.76 K, and the antiferromagnetic α‐phase at 23.88 K.^[^ ^2^, ^3^ ^]^ For the high‐pressure phases below 100 GPa, the phase diagram of oxygen was extensively investigated with the sequence of *C*2*/m* (α‐phase) *→ Fmmm* (δ‐phase at ≈6 GPa) *→ C*2*/m* (ε‐phase at ≈8 GPa) *→ C*2*/m* (ζ‐phase at ≈96 GPa).^[^ ^4^, ^5^, ^6^, ^7^, ^8^, ^9^ ^]^ As taking into account both pressure (<100 GPa) and temperature, η and η’‐phase^[^ ^10^, ^11^ ^]^ with *P*6_3_ */mmc* symmetry were identified by experiments, showing the structural complexity in oxygen. Note that the magnetic collapse takes places at the δ─ε transition whereas the insulator‐metal transition occurs at ε─ζ transition. The metallic ζ‐phase exhibits superconductivity with a transition temperature of 0.6 K.^[^ ^12^ ^]^ Most recent Raman spectra reveal that ζ‐phase may transform into other molecular phases above 175 GPa.^[^ ^13^ ^]^ As pressure further increases, the intermolecular spacing becomes comparable with the intramolecular distance, the diatomic molecules tend to become unstable and dissociate into a solid atomic state.^[^ ^14^, ^15^, ^16^, ^17^ ^]^ *Ab initio* structure searches predicted that molecular ζ‐phase transformed into a polymeric phase of O_4_ (θ‐O_4_ with I4_1_/*acd* symmetry) at ≈1.9 TPa, accompanied by the conversion from double bonds to single bonds.^[^ ^18^, ^19^ ^]^ The single bond of polymerized θ‐O_4_ is longer and weaker than the double bond of O_2_, and the lone electron pairs on the oxygen atoms result in the O_4_ spiral chain structure. Interestingly, the structure of θ‐O_4_ has also been reported in other heavier group VI elements, such as sulfur and selenium,^[^ ^20^ ^]^ illustrating the structural similarity within the same group elements. Inspired by this, the bulk sulfur and selenium with the building block of cyclic molecules S_6_^[^ ^21^, ^22^, ^23^ ^]^ and Se_6_^[^ ^24^, ^25^ ^]^ were discovered long time ago whereas the corresponding molecule O_6_ (hereinafter referred to cyclic‐O_6_) was absent to date.^[^ ^26^ ^]^ The challenge for the discovery of cyclic‐O_6_ dominantly attributes to the following reasons. First, owing to the large energy difference between single (≈142 KJ mol^−1^) and double O─O bonds (≈494 KJ mol^−1^), it takes extremely high pressure (i.e., 1.9 TPa) to convert the double bond into the single bond. Second, θ‐O_4_ was predicted to have a lower enthalpy than cyclic‐O_6_, probably preventing its formation at high pressure. Therefore, an innovative strategy must be developed to overcome this limitation.

Most recently, important progress has been made in the prediction and synthesis of cyclic‐N_6_‐based compounds. By compressing molecules N_2_ and various metals at high pressure and high temperature, the anion group of cyclic‐N_6_ was formed and stabilized by charge transfer from metal cations, such as WN_6_, TeN_6_, ReN_6_, and MoN_6_.^[^ ^27^, ^28^, ^29^, ^30^, ^31^, ^32^ ^]^ However, this strategy may be impractical to oxygen‐related systems because oxygen is inclined to promote metal oxidation rather than oxygen polymerization due to its strong chemical activity. To avoid oxidation, a more feasible strategy is to mix less inert elements (other than metal) with oxygen under high pressure. Along this track, helium appears to be a valid candidate, as it is the most inert noble gas that does not interact with other materials at ambient pressure due to the closed‐shell electronic structure. The high phase of helium with a hexagonal close‐packed structure (*P*6_3_/*mmc*) was predicted to be stable within a wide pressure range and remain in an insulating state up to ≈26 TPa.^[^ ^33^, ^34^ ^]^ However, it was established that helium can react with some molecules to form novel van der Waals (vdW) substances at high pressure.^[^ ^35^, ^36^, ^37^, ^38^, ^39^, ^40^ ^]^ For instance, the unique superionic and plastic states were predicted within helium‐water, helium‐ammonia, and helium‐methane compounds at pressure.^[^ ^38^, ^39^, ^40^ ^]^ Motivated by these encouraging reports, we herein performed an *ab initio* evolutionary search to predict a solid compound of HeO_3_ by mixing He with O_2_ above 1.9 TPa. It should be noted that the cyclic O_6_ molecules have never been found in other noble gas‐bearing materials by using the same method (Figure S1, Supporting Information), whereas the reported high‐pressure structures of Xe_3_O_2_, XeO_2_, XeO_3,_ and KrO are also absent in the He─O system.^[^ ^41^, ^42^, ^43^ ^]^ For the first time, we report a thermodynamically stable structure of HeO_3_ consisting of neutral He atoms and cyclic molecules O_6_. First‐principles calculations further reveal that helium atoms play a crucial role in reducing the reaction barrier, leading to the formation of cyclic‐O_6_ under high pressure.

## 2 Results and Discussion

**Figure 1:**
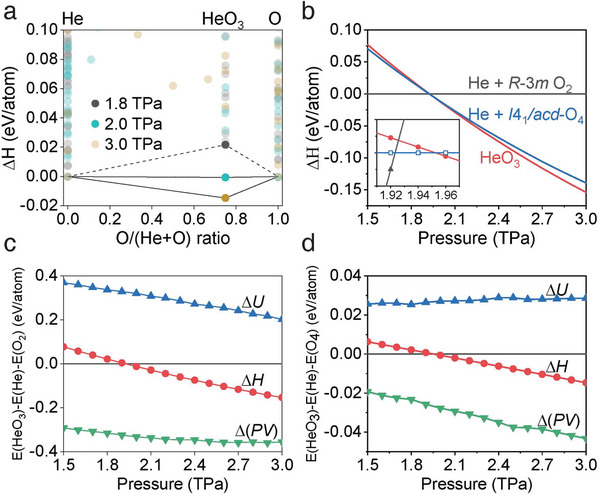
Thermodynamic stability of HeO_3_. a) Convex hull diagrams for the He─O system at selected pressures. b) The enthalpy of formation of HeO_3_ as a function of pressure with respect to hcp He and *R*‐3*m* O_2_ (black line)_,_ or hcp He and *I*4_1_/*acd* O_4_ (blue line). The inset shows an enlarged view near the phase transition pressure using hcp He and *I*4_1_/*acd* O_4_ structures for reference. c) The evolution of ∆*U* and ∆(*PV*) of HeO_3_ with respect to hcp He and *R*‐3*m* O_2_. d) The evolution of ∆*U* and ∆(*PV*) of HeO_3_ with respect to hcp He and *I*4_1_/*acd* O_4_.

**Figure 2:**
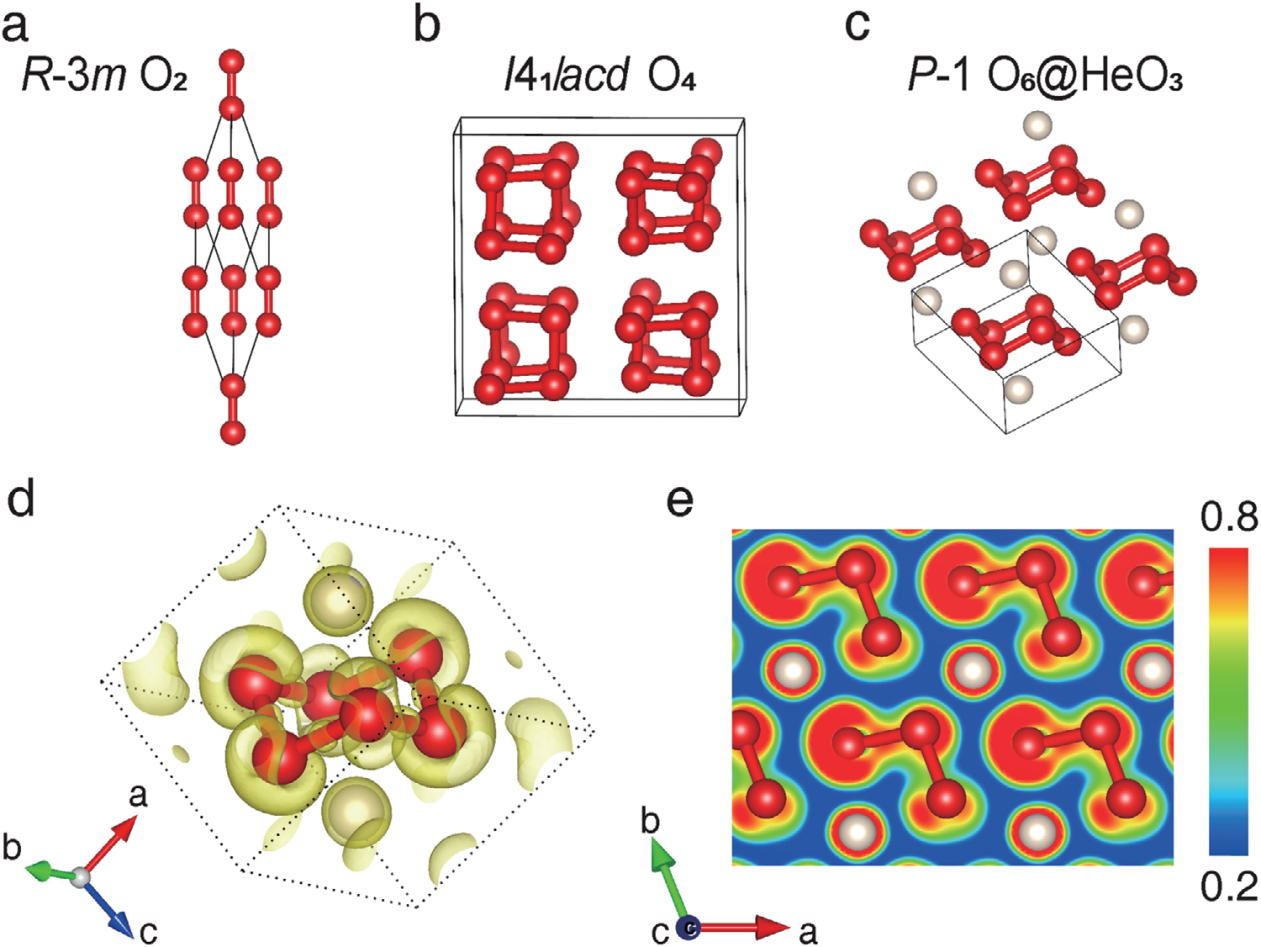
The structures of oxygen compared with HeO_3_ compound. a–c) Crystal structures of *R*‐3*m* O_2_, *I*4_1_/*acd* O_4,_ and *P*‐1 HeO_3_. d,e) 3D plotting and 2D slice of electronic localization function in HeO_3_.

First, the calculated transition pressure of 1.92 TPa from *R*‐3*m* O_2_ to θ‐O_4_ is in good agreement with previous results (Note that *R*‐3*m* O_2_ is structurally identical to ζ‐phase, see **Figures** **1**b and **2**a,b).^[^ ^18^, ^19^ ^]^ For variable‐composition evolutionary searches, the enthalpy *H* is defined as *H* = *U*+*PV*, where *U*, *P*, and *V* represent the internal energy, pressure, and volume, respectively. Thus the enthalpy of formation is defined as ∆*H* = *H*(He*_x_* O_1−_ *_x_*)−*xH*(He)−(1‐*x*)*H*(O), where *H*(He), *H*(O), and *H*(He*_x_* O_1‐_ *_x_*) are the enthalpies of solid helium, oxygen, and helium‐oxygen mixture, respectively. Accordingly, the enthalpy of *R*‐3*m* O_2_ and *P*6*_3_/mmc* He is used as a reference up to 1.9 TPa (above 1.9 TPa, the enthalpy of θ‐O_4_ was adapted accordingly). A mixture of helium and oxygen is deemed to be stable at pressure if its enthalpy of formation ∆*H* is negative. As shown in Figures 1a and S2 (Supporting Information), there is no thermodynamically stable compound between helium and oxygen up to 1.9 TPa. Strikingly, a new stoichiometry of HeO_3_ emerged on the convex hull at 2 TPa and at least persisted to 3 TPa. The inset of Figure 1b shows that both θ‐O_4_ and HeO_3_ start to become thermodynamically stable nearly at the same pressure range (i.e., ≈1.92–1.96 TPa), thus the presence of helium significantly alters the polymerization behavior of oxygen, while does not change the value of polymerization pressure. It implies that the conversion of double bonds to single bonds of oxygen determines the magnitude of polymerization pressure. To validate this hypothesis, the pressure‐dependent evolution of ∆*H*, ∆*U*, and ∆(*PV*) in HeO_3_ with respect to *P*6*_3_/mmc* He and *R*‐3*m* O_2_ were plotted in Figure 1c. It shows that the values of ∆*H* and ∆*U* dramatically decrease as the pressure increases, while ∆(*PV*) term decreases slightly (remains essentially unchanged). This suggests that the variation of internal energy ∆*U* determines the polymerization of oxygen in HeO_3_, which is related to the double‐single bonding conversion. In contrast, as the external pressure is >2 TPa and making θ‐O_4_ as the reference (Figure 1d), the values of ∆*H* and ∆(*PV*) remarkably decrease as the pressure is increased, while ∆*U* approximately remains constant. This implies that ∆(*PV*) is the leading term that triggers the formation of HeO_3_. In this case, the structure of θ‐O_4_ had completed the polymerization (Figure 2b), thus the presence of helium improves the packing efficiency and makes HeO_3_ thermodynamically stable.

The compound of HeO_3_ adopts a triclinic crystal system with space group *P*‐1. At 2 TPa, the lattice constants of HeO_3_ are *a* = 2.661 Å, *b* = *c* = 2.658 Å, α = 105.70°, β = 95.70°, and γ = 109.17°, with He atoms occupying the crystallographic sites at (0.85626, 0.76753, 0.32160) and O atoms occupying at the other sites of (0.28445, 0.76625, 0.62062), (0.28021, 0.13348, 0.18047) and (0.71221, 0.44293, 0.81503). As shown in Figure 2c, the unit cell of HeO_3_ is composed of the armchair‐like O_6_ molecule and two He atoms located above and below O_6_ center. The cyclic O_6_ contains six two‐fold coordinated oxygen atoms, being isostructural to the molecules S_6_ and Se_6_. The average O─O bond length is ≈1.13 Å, which is longer than that of O_2_ (≈1.03 Å) and slightly shorter than that of θ‐O_4_ (≈1.15 Å) at 2 TPa, suggesting its single bond nature. The shortest intermolecular distance of HeO_3_ (≈1.52 Å) is close to that of O─O between spiral chains in θ‐O_4_ (≈1.55 Å), excluding any bond between O_6_ molecules. The average O─O─O angle in O_6_ is ≈101.88°, which is almost identical to that of S_6_ and Se_6_ (≈102° at ambient pressure). In addition, the shortest distance of He─O (≈1.33 Å) and He–He (≈1.27 Å) are larger than the sum of their covalent radii, indicating no bonding formation between them. The bonding feature of HeO_3_ was also studied by calculating the electronic localization function (ELF)^[^ ^44^ ^]^ and Bader charge transfer.^[^ ^45^, ^46^ ^]^ In the computed contour plot of 3D ELF of HeO_3_, valence electrons are strongly localized along O─O bonding directions as well as around each oxygen atom, implying the presence of O─O covalent bonds and electron lone pairs (Figure 2d). Since the atomic radius of oxygen is very small at extremely high pressure, the electron lone pairs repulsion is strong, resulting in significant bond bending. Therefore, each oxygen atom in HeO_3_ has two lone pairs and two O─O covalent bonds with the bond angles of ≈101.88°. This configuration possesses a sp^3^‐like hybridization that is slightly different from the carbon atoms of cyclohexane (C_6_H_12_) with the standard sp^3^ hybridization.^[^ ^19^, ^47^ ^]^ Furthermore, no ELF basin is either observed between He and O atoms or between the neighboring O_6_ molecules, indicating the absence of strong chemical covalent bonds in this region (Figure 2e). Bader charge analysis of the total electron density shows that there is almost no ionic charge transfer between He and O atoms (≈0.01 |e|). These results indicate that HeO_3_ is a molecular compound consisting of nearly charge‐neutral He atoms and cyclic O_6_ molecules.

**Figure 3:**
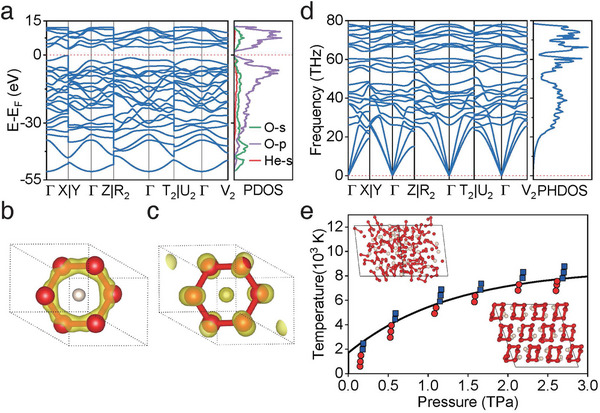
Electronic band structure, phonon properties, and thermal stability of HeO_3_ at 2 TPa. a) Band structure and projected density of states (PDOS). b) The band decomposed charge densities corresponding to energy windows of −51 to −35 eV and c) −35 to 0 eV, respectively. d) Phonon dispersion curves and projected phonon density of states (PHDOS). e) Temperature‐pressure phase diagram for HeO_3_. The red solid circle and the blue solid square represent the solid and fluid state of HeO_3_, respectively. The solid black line shows an approximate boundary for the decomposition of cyclic O_6_ molecules.

The calculated band structure and projected electronic density of states (PDOS) show that HeO_3_ is a semiconductor at 2.0 TPa with a band gap of ≈1.35 eV (**Figure** **3**a) owing to the formation of isolated O_6_ molecules and neutral He atoms. It also can be seen from Figure 3a that the deep valence bands below −35 eV originate mainly from the hybridization between the orbitals of O‐2*s* and O‐2*p*, corresponding to the contribution of O─O covalent bonds (Figure 3b). By contrast, the valence bands between −35 and 0 eV are from the dominant contributions of O‐2*p* orbitals. Within this energy range, the band decomposed charge density reveals the existence of strong electron lone pair repulsion within O_6_ molecule (Figure 3c). Figure 3d shows the phonon dispersion curve and projected phonon density of states of HeO_3_ at 2 TPa. The absence of imaginary frequencies in the whole Brillouin zone indicates that the structure is dynamically stable at 2 TPa. Additional calculations show that HeO_3_ is dynamically stable when pressure is quenched to at least 150 GPa. To further examine the thermal stability of HeO_3_, we also performed the *ab initio* molecular dynamics simulation and it shows that HeO_3_ is thermally stable ranging from 8000 K at 3 TPa and to 1500 K at 150 GPa (Figure 3e). This unveils that cyclic molecules O_6_ are persistent within a wide range of pressure and temperature as long as they are formed. As the external temperature goes beyond the melting point region (see Figure 3e), cyclic molecules O_6_ breaks into the mixture of molecular O_2_, O_3_ trimers, and some irregular intermediate O_n_ chains (n≥3). The existence of peculiar oxygen intermedia suggests cyclic molecules O_6_ may be realized at relatively low pressure if different precursors are selected, e.g., ozone or O_3_ trimers (Figure S3, Supporting Information), provided that they are stable under low pressure.^[^ ^48^ ^]^

For the exotic compound HeO_3_, it is also interesting to study the stability of cyclic O_6_ when the helium atoms are removed. Using the same methods, phonon dispersion curves show that pure cyclic O_6_ molecules are dynamically stable at a pressure ranging from 150 GPa to 2 TPa. To investigate the structure difference between O_6_@HeO_3_ and He‐removed O_6_ structure, **Table** **1** listed their optimized bond lengths, bond angles, and dihedral angles at 2 TPa. As we see, the bond angles and dihedral angles of O_6_@HeO_3_ are very close to those of S_6_ and Se_6_ at ambient pressure except for the bond lengths (note that 2 TPa for HeO_3_ while zero pressure for S_6_ and Se_6_). This indicates that O_6_@HeO_3_ has almost the same structural configuration as S_6_ or Se_6_. However, for the He‐removed O_6_ structure, there is a significant deviation in their bond angles and dihedral angles compared with that of S_6_ and Se_6_. Specifically, it is ≈9% and 7% smaller in the bond angle while 17% and 13% larger in the dihedral angle than that of S_6_ and Se_6_, respectively. This suggests that He atoms play an important role in controlling the configuration of cyclic O_6_ and thus stabilizing them by reducing the electron lone pair repulsion.

**Table 1:** The average bond length, bond angle, and dihedral angle of S_6_, Se_6_ at ambient pressure compared with those of cyclic O_6_ in HeO_3_ and He‐removed O_6_ at 2 TPa.

Structure	Bond length [Å]	Bond angle [°]	Dihedral angle [°]
S_6_	2.08	102.9	73.8
Se_6_	2.36	101.1	76.2
O_6_@HeO_3_	1.13	101.8	77.6
He‐removed O_6_	1.15	93.9	86.1

**Figure 4:**
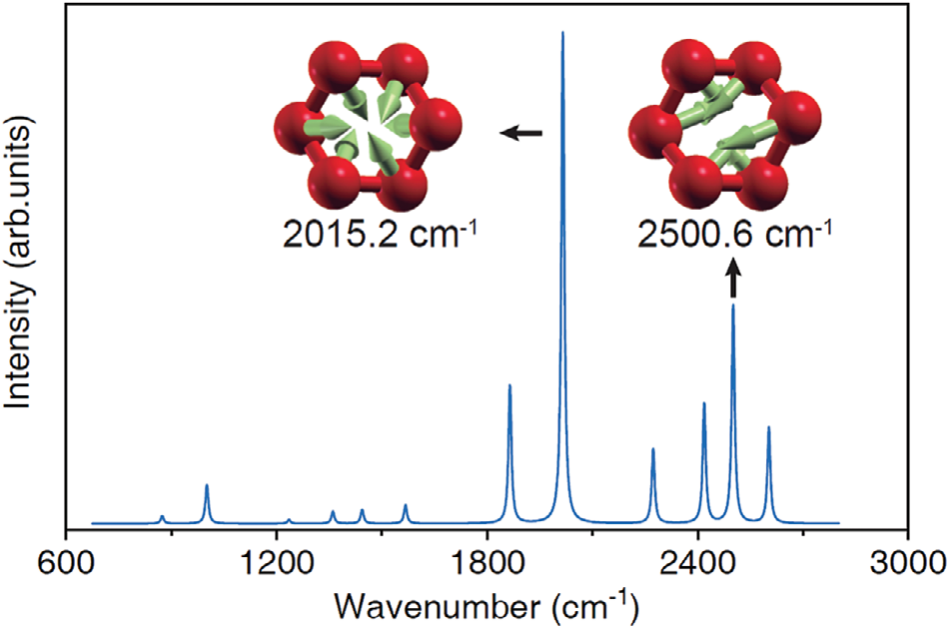
Calculated Raman spectrum of HeO_3_ at 2 TPa. The inset shows the eigenvectors of the two strongest peaks, in which green arrows indicate the direction and magnitude of oxygen displacements in the O_6_ rings. For clarity, the displacements of helium atoms are not shown.

Owning to the unique presence of the O_6_ rings, it is natural to question if these rings would lead to a different vibrational spectrum in this remarkable HeO_3_ phase. **Figure** **4** shows the simulated Raman spectrum and vibration modes of HeO_3_ at 2 TPa. Factor group analysis of *P*‐1 HeO_3_ reveals that 24 zone center vibrational modes can be assigned as Γ = 12A_g_+12A_u_. There are 12 Raman‐active modes with A_g_ symmetry plotted in Figure 4. The relatively strong Raman peaks are 1864.7, 2015.2, 2272.8, 2418.2, 2500.6, and 2602.2 cm^−1^, respectively. By carefully checking the eigenvectors of these vibration modes, the results show that the two strongest peaks of 2015.2 and 2500.6 cm^−1^ attributed to the symmetric stretching vibrations whereas others are asymmetric stretching vibrations. These 6 characteristic vibration modes may serve as the fingerprint of cyclic O_6_ molecules for future experimental detection.

**Figure 5:**
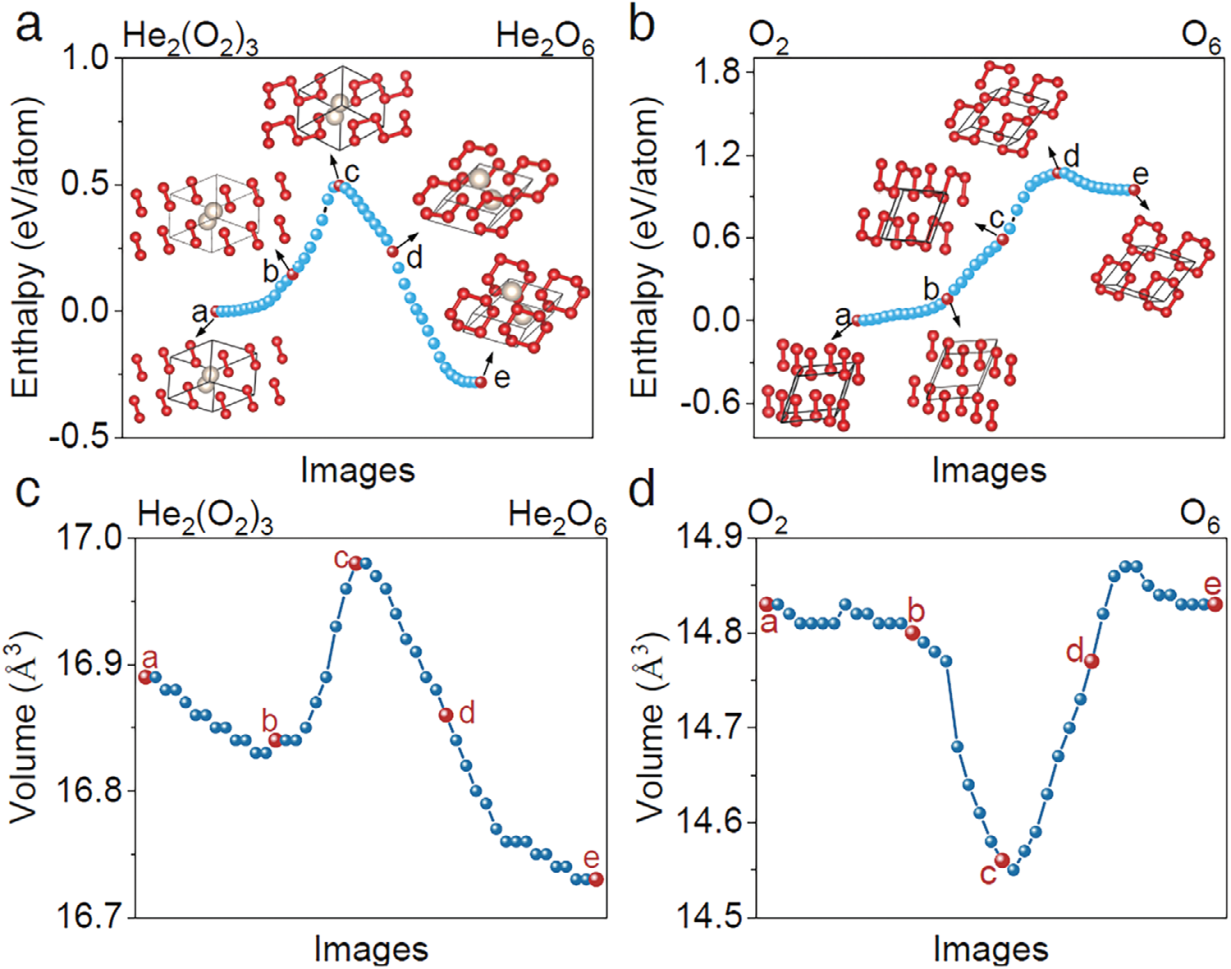
Transformation pathways of HeO_3_ at 2 TPa. a) The phase transition path from He_2_(O_2_)_3_ to HeO_3_ and b) from *R*‐3*m* O_2_ to He‐removed O_6_. c,d) The corresponding volume evolution of (a) and (b). The letters a, b, c, d, and e represent the typical transition states accordingly.

To understand the formation mechanism of HeO_3_ and the role of helium atoms, we also investigated the energetically favorable transition pathways both from *R*‐3*m* O_2_ to O_6_ and from a hypothetic structure of He_2_(O_2_)_3_ (by adding He atoms to the lattice voids in *R*‐3*m* O_2_) to HeO_3_ with the variable cell nudged elastic band (VCNEB) method.^[^ ^49^, ^50^ ^]^ The phase transition paths and the characteristic atomistic snapshots are summarized in **Figure** **5**a,b. At 2 TPa, regardless of whether helium atoms exist or not, the transformation processes are generally classified into four stages. The first stage *a*‐*b*, mainly involves the rotation of molecular O_2_. Even different initial structures are adopted, through the translation and rotation of O_2_, it ultimately provides a favorable environment for the formation of intermediate products. For the second stage *b‐c*, each of three neighboring O_2_ molecules is polymerized to form a zigzag intermediate O_6_ cluster that mimics the digital 2 shape, followed by a decomposition to two O_3_ trimers at the third stage *c‐d*. At the final stage *d‐e*, the intermedia O_3_ transforms into the cyclic molecule O_6_ at high pressure. For the phase transformation from He_2_(O_2_)_3_ to HeO_3_, stages *a‐c* determine the height of the energy barrier (≈0.5 eV/atom at 2 TPa in Figure 5a) while the leading energy barrier from O_2_ to helium‐removed O_6_ is determined by stage *a‐d* (≈1 eV/atom at 2 TPa in Figure 5b). Additionally, Figures 5c and 5d show the corresponding volume variation of transition states which exhibit a completely different evolutionary behavior during the critical transformation stage. For instance, the presence of helium results in volume expansion of He_2_(O_2_)_3_ at *b‐c* stage, which alleviates the Coulomb repulsion between O_2_ and promotes the formation of intermedia O_6_. Once the reaction barrier is crossed, the volume decreases at stages *c‐d* and *d‐e*, and then the intermediate O_6_ finally polymerizes into the denser O_6_. Therefore, despite that the atomistic transformation mechanism from *R*‐3*m* O_2_ to O_6_ is quite similar to that of He_2_(O_2_)_3_ *→* HeO_3_ transition, their volume profiles are notably distinct, thus leading to very different energy barrier values. These results suggest that the addition of helium is a key to achieving the cyclic O_6_ molecules. Without helium, cyclic molecules O_6_ may not be directly synthesized from pure molecular O_2_ even under a very high pressure.

## 3 Conclusion

In summary, we systematically study helium‐oxygen mixtures at the multi‐TPa regime using *ab initio* evolutionary searches. According to our study, there is only one stable compound, HeO_3_, consisting of neutral He atoms and hexameric molecules O_6_. This exotic molecular phase is thermodynamically stable above 1.9 TPa as well as dynamically stable down to 150 GPa. It may be formed at pressures accessible to ramp compression experiments, as exemplified by the U.S. National Ignition Facility (up to ≈5 TPa),^[^ ^51^ ^]^ or synthesized at much lower pressure and high temperature, for instance, the formation of Fe─He compounds at high pressure.^[^ ^52^, ^53^ ^]^ In addition, since helium and oxygen are two of the most abundant elements in the solar system, their mixtures, particularly for the high‐pressure form of molecules O_6_, are helpful in understanding the relationship between structures and properties of outer planetary interiors, such as Saturn and Jupiter.

## 4 Experimental Section

The variable‐composition evolutionary algorithm (USPEX)^[^ ^54^, ^55^ ^]^ combined with density functional theory (DFT) calculations was utilized to explore thermodynamically stable He─O compounds under the multi‐TPa range. Structure relaxation and electronic properties were calculated within the generalized gradient approximation (GGA) using the Perdew‐Burke‐Ernzerhof (PBE)^[^ ^56^ ^]^ functional, as implemented in the VASP code.^[^ ^57^ ^]^ More details of the theoretical calculations are listed in the Supporting Information.

## Conflict of Interest

The authors declare no conflict of interest.

## Supporting information



Supporting Information

## Data Availability

The data that support the findings of this study are available from the corresponding author upon reasonable request.
