# Supplementary material for: Prediction of Cyclic O6 Molecules Stabilized by Helium under Pressure
Source: Adv Sci (Weinh). 2025 Jan 24;12(11):2415517. doi: 10.1002/advs.202415517 (PMC11923858; doi:10.1002/advs.202415517)
Supplement: Supplementary file 1 — Supporting Information [file ADVS-12-2415517-s001.docx]

**Supporting Information**

**Prediction of Cyclic O_6_ Molecules Stabilized by Helium under Pressure**

*Jingyu Hou, Qiang Zhu, Xiao-Ji Weng, Xi Shao, Xiao Dong, Hui-Tian Wang, Xiang-Feng Zhou^*^, and Yongjun Tian*

**Calculation details**

The variable-composition evolutionary algorithm (USPEX)^[1,2]^ combined with density functional theory (DFT) calculations was utilized to explore thermodynamically stable He-O compounds under the multi-TPa range. Specifically, structure searches were conducted at pressures of 0.2, 0.5, 1.0, 1.5, 1.8, 2.0, 2.5, and 3.0 TPa, respectively. To achieve an unbiased sampling of the entire composition range, simultaneous variations in structures and stoichiometries were implemented for structure searches. Each search involved up to a maximum of 40 atoms per primitive cell and ran for 50 generations with 60 structures per generation. For the first generation, structures were completely randomized and subsequent generations followed a distribution of 20% by heredity, 20% by lattice mutation, 10% by transmutation, and 50% by newly added random structures. The total number of structures under each search amounted to approximately 3000. Structure relaxation and electronic properties were calculated within the generalized gradient approximation (GGA) using the Perdew-Burke-Ernzerhof (PBE)^[3]^ functional, as implemented in the VASP code.^[4]^ Due to the extreme pressure involved in structure searches and calculation of properties, we used very HARD projector augmented wave (PAW) pseudopotentials^[5]^ for both He and O with the cutoff radii less than 0.6 and 1.2 Bohr, respectively. A cutoff energy of 2800 eV and uniform Γ-centered *k* meshes with a resolution of 2π×0.06 Å^−1^ were chosen for structure searches and denser *k* meshes (2π×0.03 Å^−1^) were applied to calculate energy and property, e.g., ensuring the convergence of enthalpy and force better than 10^-7^ eV and 10^-3^ eV/Å, respectively. To validate the thermal and dynamical stability of the predicted structure, the *ab initio* molecular dynamics (AIMD) simulations were conducted using a canonical NVT ensemble with Nosé–Hoover thermostat.^[6]^ The 3×3×3 supercell, energy cutoff of 1000 eV, and Γ-only *k*-mesh were employed for AIMD simulations. Each simulation lasted for 10 ps with the time step of 1 fs. Within the framework of density functional perturbation theory (DFPT), Phonon dispersion curves were computed from the PHONOPY^[7]^ package and Raman spectrum were simulated using the Fonari-Stauffer method.^[8]^ We also calculated the electronic properties and dynamic stability of He-O system using the semiempirical dispersion corrected optB88-vdW method.^[9]^ The corresponding results are in good agreement with those from the PBE functional, implying the vdW interaction is not significant for the electronic structure at the multi-TPa regime.

Besides He-O system, we also explored the possibility of other noble gas reacting with oxygen to form the cyclic O_6_ molecules (Figure S1). However, there is no thermodynamically stable compounds in the Ne-O system up to 5 TPa, which may attribute to the large atomic size and the varied reactivity under pressure.^[10]^ By contrast, a series of noble gas oxides were predicted to be stable among Ar-O, Kr-O and Xe-O systems, which is consistent with the reported results.^[11-13]^ Owing to the formation of chemical bonds between oxygen and most of noble gas atoms, it excludes the possibility of formation of cyclic O_6_ rings in a noble gas element other than helium.

Reference

1. A. R. Oganov, C. W. Glass, J. Chem. Phys. 2006, 124, 244704.
2. A. O. Lyakhov, A. R. Oganov, H. T. Stokes, Q. Zhu, *Comput. Phys. Commun.* **2013**, *184*, 1172.
3. J. P. Perdew, K. Burke, M. Ernzerhof, *Phys. Rev. Lett.* **1996**, *77*, 3865.
4. G. Kresse, J. Furthmuller, *Phys. Rev. B* **1996**, *54*, 11169.
5. P. E. Blöchl, *Phys. Rev. B* **1994**, *50*, 17953.
6. W. G. Hoover, *Phys. Rev. A* **1985**, *31*, 1695.
7. A. Togo, I. Tanaka, *Scr. Mater.* **2015**, *108*, 1.
8. A. Fonari, S. Stauffer, vasp_raman.py. can be found under https://github.com/raman-sc/VASP/, **2013**.
9. J. Klimeš, D. R. Bowler, A. Michaelides, *J. Phys. Condens. Matter* **2009**, *22*, 022201.
10. X. Dong, A. R. Oganov, H. Cui, X. F. Zhou, H. T. Wang, Proc. Natl. Acad. Sci. USA. 2022, 119, e2117416119.
11. Q. Zhu, D. Y. Jung, A. R. Oganov, C. W. Glass, C. Gatti, A. O. Lyakhov, Nat. chem. 2013, 5, 61.
12. A. Dewaele, N. Worth, C. J. Pickard, R. J. Needs, S. Pascarelli, O. Mathon, M. Mezouar, T. Irifune, Nat. chem. 2016, 8, 784.
13. P. Zaleski-Ejgierd, P. M. Lata, Sci. Rep. 2016, 6,18938.


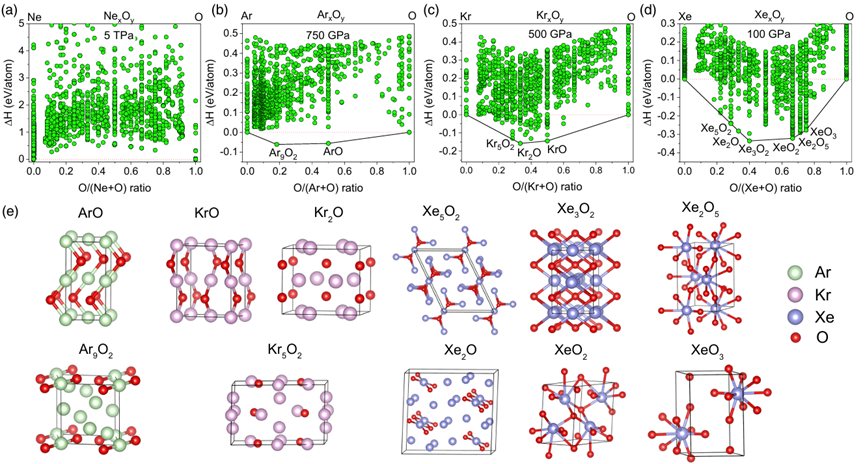


**Figure S1.** Predicted convex hulls for the (a) Ne-O system at 5.0 TPa, (b) Ar-O stsyem at 750 GPa, (c) Kr-O system at 500 GPa, and (d) Xe-O system at 100 GPa. Each solid circle in the figures represents a structure, and part of low-energy structures is shown accordingly. (e) The stable structures of Ar-O, Kr-O, and Xe-O system. Note that structure searches of Ne-O, Ar-O, and Kr-O system were also performed at lower pressures but no stable components were found.


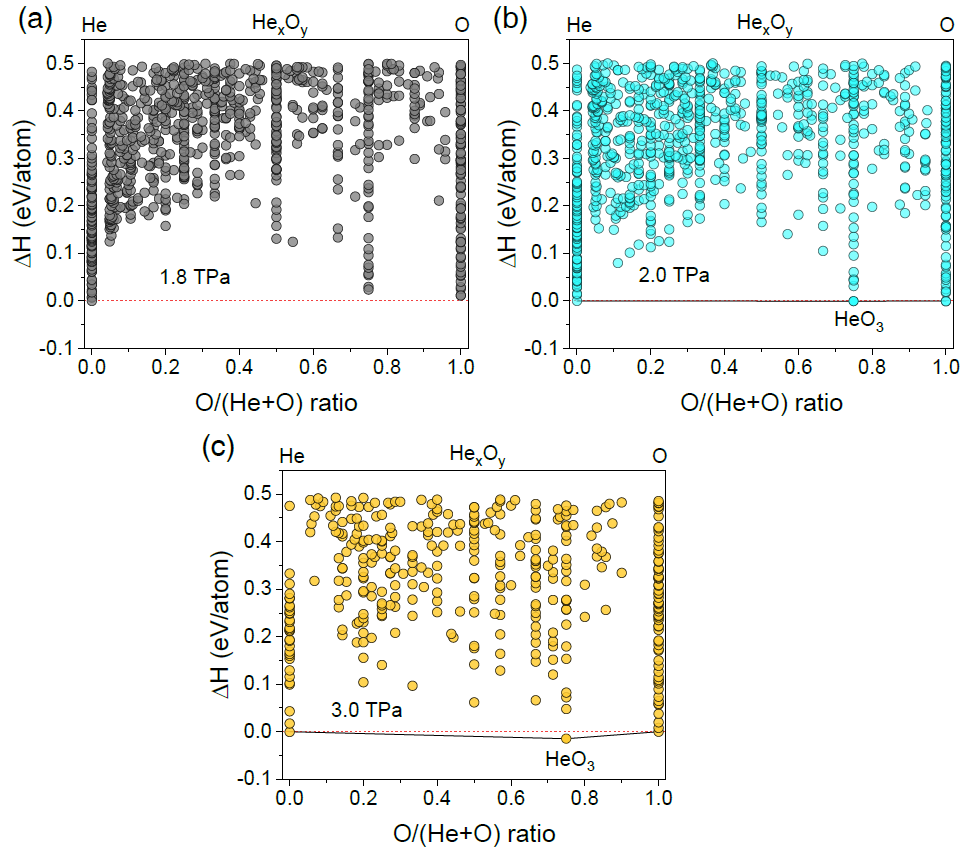


**Figure S2.** Enthalpies of formation for the He-O system at pressures of (a) 1.8 TPa, (b) 2.0 TPa, (c) 3.0 TPa. There is only a stable phase of HeO_3_ at 2.0 and 3.0 TPa. Each solid circle in the figures represents a structure, and the variable-composition searches include a considerable number of components. Note that Fig. R1 is a part of Fig.1a in the manuscript, and only part of structures was shown in Fig.1a within the enthalpy of formation ranged from -0.02 to 0.10.


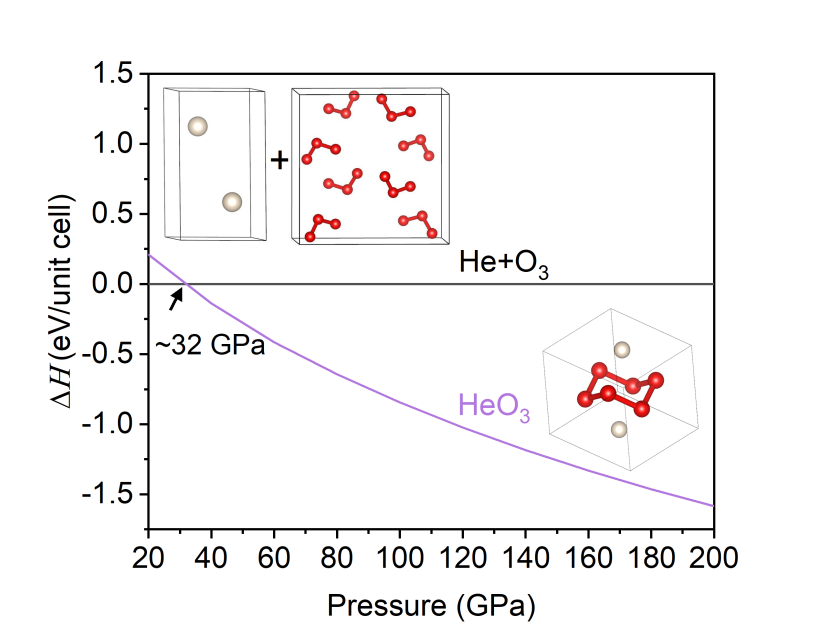


**Figure S3**. The enthalpy of formation of HeO_3_ as a function of pressure with respect to hcp He and *Pbca* O_3._
